# Independent origins of neurons and synapses: insights from ctenophores

**DOI:** 10.1098/rstb.2015.0041

**Published:** 2016-01-05

**Authors:** Leonid L. Moroz, Andrea B. Kohn

**Affiliations:** 1 The Whitney Laboratory for Marine Bioscience, 9505 Ocean Shore Boulevard, St Augustine, FL 32080, USA; 2 Department of Neuroscience and McKnight Brain Institute, University of Florida, Gainesville, FL 32611, USA

**Keywords:** Ctenophora, synapses, Cnidaria, Porifera, Placozoa, nervous system evolution

## Abstract

There is more than one way to develop neuronal complexity, and animals frequently use different molecular toolkits to achieve similar functional outcomes. Genomics and metabolomics data from basal metazoans suggest that neural signalling evolved independently in ctenophores and cnidarians/bilaterians. This polygenesis hypothesis explains the lack of pan-neuronal and pan-synaptic genes across metazoans, including remarkable examples of lineage-specific evolution of neurogenic and signalling molecules as well as synaptic components. Sponges and placozoans are two lineages without neural and muscular systems. The possibility of secondary loss of neurons and synapses in the Porifera/Placozoa clades is a highly unlikely and less parsimonious scenario. We conclude that acetylcholine, serotonin, histamine, dopamine, octopamine and gamma-aminobutyric acid (GABA) were recruited as transmitters in the neural systems in cnidarian and bilaterian lineages. By contrast, ctenophores independently evolved numerous secretory peptides, indicating extensive adaptations within the clade and suggesting that early neural systems might be peptidergic. Comparative analysis of glutamate signalling also shows numerous lineage-specific innovations, implying the extensive use of this ubiquitous metabolite and intercellular messenger over the course of convergent and parallel evolution of mechanisms of intercellular communication. Therefore: (i) we view a neuron as a functional character but not a genetic character, and (ii) any given neural system cannot be considered as a single character because it is composed of different cell lineages with distinct genealogies, origins and evolutionary histories. Thus, when reconstructing the evolution of nervous systems, we ought to start with the identification of particular cell lineages by establishing distant neural homologies or examples of convergent evolution. In a corollary of the hypothesis of the independent origins of neurons, our analyses suggest that both electrical and chemical synapses evolved more than once.

## 1. Introduction

Nervous systems are incredibly diverse in both their cellular composition and numerical context [1]. The numbers of neurons range from a few hundred in nematodes and rotifers to 86 billion and 257 billion in humans [2] and elephants [3], respectively. Regardless of the species, neurons come in many varieties, sometimes with thousands of cellular subtypes, and their classification is not established, particularly across phyla [4–6]. Even in well-characterized simpler neural circuits or mammalian brain regions, the spectrum of anticipated neuronal diversity is largely unknown [4,7–12]. Unsurprisingly, the long-standing questions about the origins and evolution of neural systems are subjects of ongoing controversies [1,13–26].

Could neurons evolve more than once? Both a single origin and multiple origins (polygenesis) have been discussed in the literature, but no consensus has been achieved (see details in [13,14]). Historically, the hypothesis of the single origin had become the predominant point of view and was virtually unquestioned until very recently [13,27,28]. Several factors contributed to this situation, including the old postulate that Porifera (or sponges) represent the descendants of the earliest animal lineage. As such, starting with Parker's famous hypothesis [16], Porifera were considered to represent the pre-neuronal ‘stage’ of evolution. The second ‘stage’ was represented by Cnidaria, with their diffuse nerve net and the possibility of a single neuronal origin from the conductive myoepithelial cells in ancestral cnidarians [23,26]. Ctenophores and placozoans were largely ignored in such evolutionary reconstructions. However, in the classical zoology literature, placozoans (*Trichoplax adhaerens*) were considered to represent one of the simplest grades of animal organization, sometimes ‘competing’ with sponges for the most basal position at the animal tree of life [29–31]. By contrast, ctenophores were often united with Cnidaria into the clade Coelenterata [32]. The textbook consensus was that the central nervous system also evolved only once [33–36]. This simplified perception of a gradual increase in neuronal complexity existed without any radical changes for more than 100 years. The genomic revolution of the twenty-first century changed this situation dramatically. Even the initial cladistic analysis suggested that neurons and central nervous systems may have evolved more than once from genealogically different cell lineages (see details in [13,27]).

When considering the arguments ‘for’ and ‘against’ the different evolutionary scenarios, two major factors must be critically evaluated: (i) the progress towards an unbiased reconstruction of the animal phylogeny, and (ii) the criteria for establishing homology versus analogy/homoplasy in the identification of complex characteristics or traits, such as neurons, synapses, neural circuits and brain regions. In general, the lack of *uniquely shared* molecular components or mechanisms between morphologically or functionally similar traits (e.g. eyes, muscles, neurons or synapses) is indicative of convergent evolution or homoplasy. Homoplasy implies that a given trait was not present in the common ancestor. For example, the urmetazoan lacked striated muscles and eyes, and these characteristics evolved independently in multiple animal groups [37,38]. In fact, it is now widely accepted that eyes evolved more than 30–40 times [38], and the superficial similarity of the complex camera eyes in cephalopods and vertebrates represents the classical case of convergent evolution [39].

To support or reject the hypothesis of a single origin of a nervous system, we must first find the uniquely shared molecular traits for all neurons and synapses. Do pan-neuronal and pan-synaptic genes/molecular markers exist? The most recent analysis has suggested that this is not the case [40], implying that neuronal phenotypes are results of convergent evolution. For example, neuronal phenotypes with synapses might have evolved independently in different lineages, such as those leading to snails and comb jellies. Any quest for deep neuronal genealogies requires the establishment of the evolutionary relationships among basal metazoans. By itself, the animal classification is highly controversial and an active field of research, but novel phylogenomics tools have already resolved several key nodes in the animal tree of life [41–44]. These advances, together with genomic studies on enigmatic neural systems, challenge the existing zoology and neuroscience textbooks. Here, we will outline the recent progress suggesting independent origins and parallel evolution of neural systems. The first and most debated question is: What are the first two branches of multicellular animals?

## 2. Ctenophores as the sister group to all other metazoans

The quest to establish the relationships of the animal lineages (Bilateria, Cnidaria, Porifera, Placozoa and Ctenophora) has puzzled biologists for centuries [44]. Until very recently, the most commonly accepted view was that Porifera (sponges) are the sister to all extant animals. This view was also consistent with the relative morphological simplicity of sponges, including the absence of recognized neurons and muscles.

**Figure 1.**
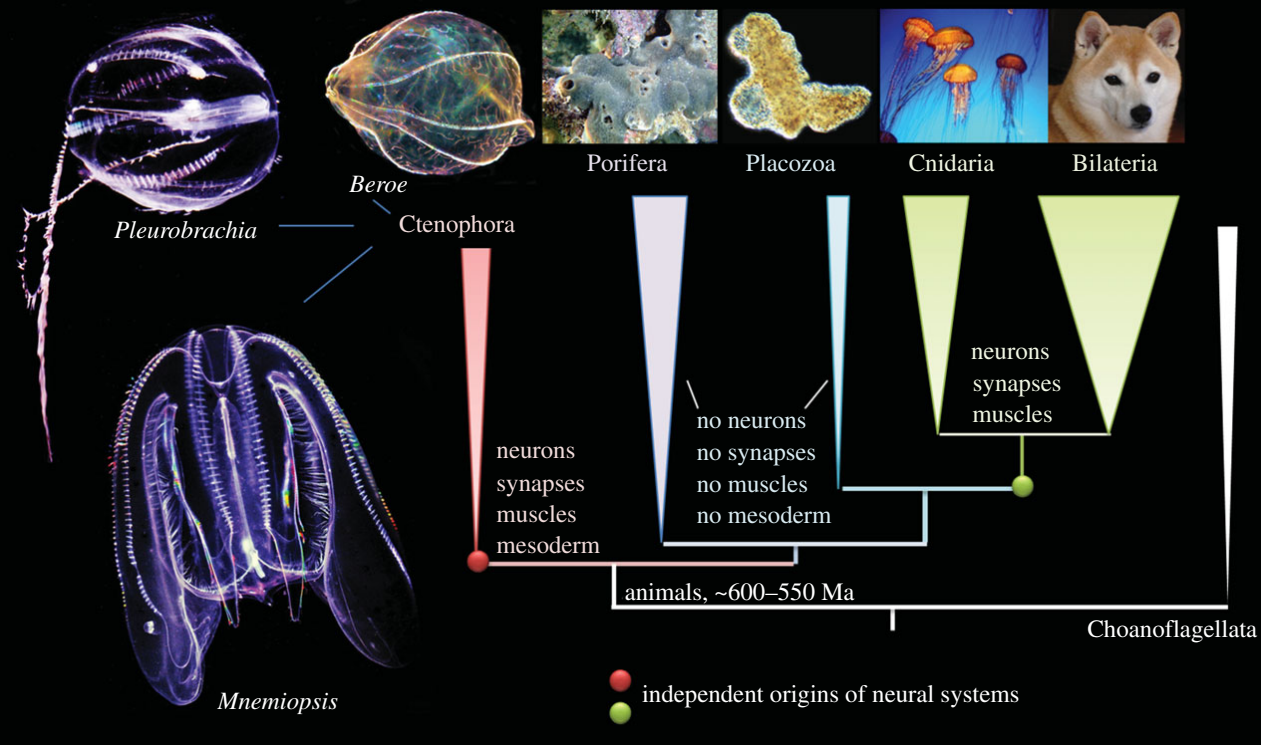
Relationships among five basal metazoan clades with choanoflagellates sister to all animals. Three ctenophore species are shown on the left. The sea gooseberry, *Pleurobrachia bachei* and the sea walnut, *Mnemiopsis leidyi* are two ctenophores with their genome recently sequenced. The most recent phylogenomics and comparative analyses suggest convergent evolution of neurons, synapses, muscles and mesoderm in Metazoa. Here, we also stress three major points: (i) neuron is a functional, but not a genetic category; (ii) any given neural system is not a single character, it includes different cell lineages with different genealogies and origins; and (iii) there are no pan-neuronal/pan-synaptic genes. The figure is modified from Moroz *et al*. [28]. Photos: L.L. Moroz. See text for details.

An initial challenge to the Porifera-first hypothesis was provided by Dunn *et al*. [45]. Their matrixes for phylogenomic analysis included expressed sequence tag (EST) data from 77 taxa, of which 71 were from 21 animal phyla, with approximately 44.5% overall matrix completeness. One of the outcomes of this analysis was the hint that the comb jellies (represented by *Mnemiopsis* and an undescribed mertensiid species) form a sister group to all other sampled metazoans. However, Dunn *et al*. stated that this hypothesis ‘should be viewed as provisional until more data are considered from placozoans and additional sponges’. Indeed, the *Trichoplax* data were not included in the analysis, and sponges were represented by only two species (*Oscarella carmela* and *Suberites domuncula*). Moreover, in both summary trees (figs 1 and 2 in Dunn *et al*.) *sponges were placed as sister to cnidarians* [45]. In addition, the support for the now well-established clades Ecdysozoa and Lophotrochozoa was also limited.

The subsequent phylogenomic analysis by some of the authors [46] expanded the list of species. Specifically, Hejnol *et al*. [46] included ESTs from one additional sponge, *Amphimedon queenslandica,* and emerging genomic data from *T. adhaerens* (Placozoa). Both the 844- and 330-gene matrixes were analysed and supported the placement of ctenophores as the sister group to all remaining animals. Surprisingly, sponges were recovered as a polyphyletic, and *Trichoplax* was placed within the clade of Porifera, a position that contradicts that of many other studies [30,47]. Support for Lophotrochozoa or Spiralia was also low, and the relationships within this group were mostly unresolved. Unsurprisingly, the authors remained cautious and stressed that these results ‘should be treated provisionally’ until new EST and genome projects from sponges and ctenophores became available and were ‘rigorously evaluated’.

The follow-up phylogenetic studies (2009–2013) with the sequenced sponge [30] and placozoan [29] genomes and the comparative data from next-generation sequencing (RNA-seq/transcriptomes) added even more controversies [47,48]. The conflicting hypotheses included the re-establishment of sponges as the sister to all animals and a ‘recovery’ of ctenophores as the sister group to Cnidaria (i.e. the classical Coelenterata hypothesis [32,47]). The placement of Placozoa as the sister to all other animals was also considered [31]. Finally, the systematic review of all 35 animal phyla led Nielson [49] to place ctenophores as the sister to bilaterian animals [49] owing to their well-developed neuromuscular systems and mesoderm and complex tissue organization. A comparison of sequenced mitochondrial genomes, including ctenophores [50,51], provided little help because of their highly derivative nature. In most of the analyses performed at that time, the ctenophore data were very limited and fuelled the ongoing controversies. Thus, the sequencing of the ctenophore genomes was a critical step to address the inherent phylogenomic challenges arising from the incomplete transcriptome datasets.

Two groups of investigators independently initiated ctenophore genome sequencing projects using the cydippid (*Pleurobrachia bachei*) and the lobate (*Mnemiopsis leidyi*). The results of the *Pleurobrachia* whole-genome sequencing were formally reported at the Society for Integrative and Comparative Biology (SICB) meeting in Charleston (SC) in January 2012 [52] and suggested the ctenophore-first hypothesis and convergent evolution of ctenophore neural systems. A more extensive analysis was presented at the SICB meeting in San Francisco in 2013, further supporting the position of ctenophores as the sister group to all other animals [28,53,54]. In addition, we performed deep transcriptome sequencing using other ctenophore species (*Euplokamis dunlapae, Coeloplana astericola, Vallicula multiformis, Pleurobrachia pileus, Dryodora glandiformis, Beroe abyssicola, Bolinopsis infundibulum,* an undescribed mertensid and *M. leidyi*), which allowed the comparative validation of the initial predictions from the ctenophore genomes and resolved the internal ctenophore phylogeny [28].

Meanwhile, a *Mnemiopsis* sequencing project was initiated and performed at the NIH by the Baxevanis team [55,56] and was reported at the same San Francisco meeting, but the ctenophore-first hypothesis was not confirmed [57]. However, it was difficult to interpret the genomic data without a strong phylogenomic context. Eventually, the *Mnemiopsis* consortium also came to the same conclusion as the *Pleurobrachia* team [28,52,55]; both genome-wide studies recognized Ctenophora as the earliest branching animal lineage and sister to all metazoans (figure 1).

Nevertheless, this conclusion was not fully accepted by the comparative community [57,58] because of the limited amount of available comparative data and the complexity of the statistical analyses used in phylogenomic studies. For example, the NIGRI consortium produced a summary tree using maximum-likelihood analysis of gene content [55]. In this tree, Ctenophora, represented by *Mnemiopsis*, was the most basal animal lineage. However, with the same 100% bootstrap support, molluscs (*Lottia*) and annelids (*Capitella*) were incorporated into the chordates (see fig. 4 in [55]). This situation was similar to earlier large-scale phylogenomic studies, and resolving one branch frequently led to conflicting placements of other lineages (see above; [32,45,46]). In the *Pleurobrachia* genome paper, we obtained 100% bootstrap and statistical support for the Ctenophora-first hypothesis (*Pleurobrachia* + *Mnemiopsis*) and other major metazoan branches. Similar results were also obtained using different datasets and methods [59]. However, the support for the earliest branching of ctenophores was reduced when we added the RNA-seq data from 10 ctenophore species (see figure 3 and extended data in [28]).

To resolve these potential controversies, Whelan *et al*. [60] performed systematic analyses of the errors and signals from all available datasets, including genome-scale data from 10 ctenophore species, as well as extensive sponge, placozoan and cnidarian transcriptomes. As a result, the most recent phylogenetic analysis strongly supports the placement of ctenophores as the sister group to all animals and has 100% support for all other basal metazoan lineages [60]. It is the most complete and unbiased study available to date (figure 1). Nevertheless, additional comparative genomic data, particularly from other lineages of sponges and ctenophores, would be indispensable for understanding the deep metazoan phylogeny. Table 1 and the electronic supplementary material, table S1 summarize the major groups of genes identified in the sequenced ctenophore genomes that are relevant to the questions of the origins and evolutions of neural systems discussed below.

**Table 1.** Representation of different families (number of genes) in the genomes *Pleurobrachia* and *Mnemiopsis.*

gene/family	*Pleurobrachia*	*Mnemiopsis*
**ion channels/receptors**		
voltage-gated potassium channels	36	44
potassium channels	17	16
voltage-gated calcium channels	1	1
two pore calcium channel	3	3
cation channels of sperm (CatSper)	6	1
voltage-gated sodium channels	2	2
sodium leak channel (NALCN)^a^	0	0
voltage-gated proton channel (H^+^)	1	1
HCN^a^/cyclic nucleotide-gated ion channel (CNG)	0/3	0/3
transient receptor potential channel (Trp)	13	13
calcium release activated channel (ORAI)	1	1
ENaC/ASSC channel^b^	29	28
ionotropic glutamate receptor (iGluR)^b^	14	14
P2X receptors	1	1
seven transmembrane receptors^b^	697	567
**electrical signalling**		
pannexin/innexin^b^	12	12
**glutamate signalling**		
sialin-like transporters^b^	8	9
glutaminases^b^	8	9
glutamate decarboxylase	1	1
excitatory amino acid transporter	1	1
**gaseous signalling**		
nitric oxide synthase (NOS)^a^	0	1
soluble guanylyl cyclase	4	3
haem oxygenase	1	1
cystathionase	1	1
**peptidergic signalling**		
secretory peptides (0.8 cut-off)	351	375
secretory peptides (0.9 cut-off)	72	42
pore-forming toxins^b^	9	12
**signalling**		
Wnt	4	4
**enzymes**		
serine racemase	1	0
arginine kinase	4	0
**RNA binding**		
RNA-binding proteins	269	269
ELAV	3	4
Nanos	1	2
NOVA	>15	>15
**small RNA processing**		
argonaute	4	4
dicer	1	1
Piwi	3	3
HEN1 methyltransferase	1	1
**extracellular matrix**		
collagen IV^b^	7	14
integrin	26	16
**synaptic signalling/exocytosis**		
synaptobrevin	1	1
synaptojanin	1	1
syntaxin	3	3
epsin	1	2
synaptotagmin	3	3
complexin	1	1
neurexin	1	1
neuroligin	0	0
**epigenetic regulation** (see [61,62])		
DNA methyltransferase 1 (DNMT1)	1	1
6-mA methyltransferase 4-like	1	1
6-mA demethylase-like	1	1
RNA editing enzymes^b^	14	10

^a^Secondary gene loss from the common metazoan ancestors.

^b^Ctenophore lineage-specific expansion of a given gene family.

## 3. Polygenesis versus single origin: comparison of the two alternative hypotheses of neural evolution supports the independent origin of neurons in ctenophores

**Figure 2.**
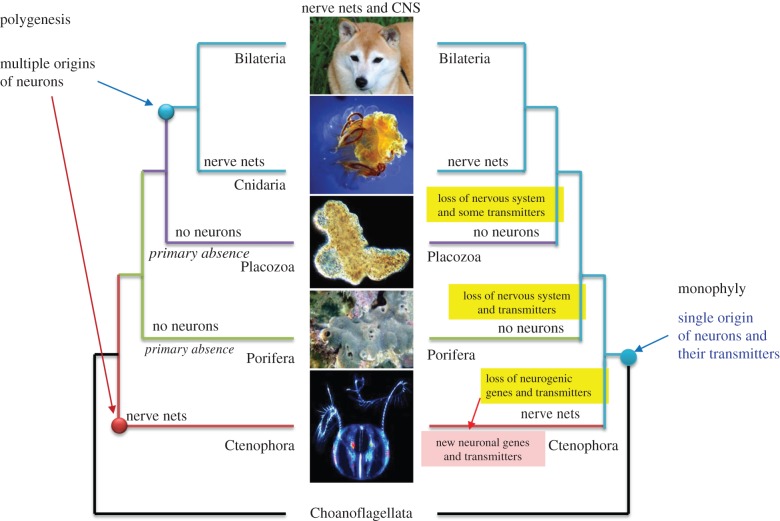
Two alternative scenarios of neuronal evolution (modified from Moroz *et al*. [14]). The polygenesis or multiple origins of neurons as the example of convergent evolution (left). The single-origin hypotheses implies multiple loss of neural systems in sponges and placozoans as well as massive loss of many molecular components involved in neurogenesis and synaptic functions of the Urmetazoan in ctenophores (right). The single-origin hypothesis still implies independent recruitment of other molecular components involved in neural and synaptic functions—the situation which still suggests the extensive parallel evolution of neural organization in ctenophores. Here, ctenophores are placed as sister to other animals. However, even the classical view of the animal phylogeny (sponges are sisters to other animals, see fig. 2 in [14]) still implies the parallel evolution of neurons and neural signalling in the animal kingdom. See text for details.

Although earlier phylogenomic papers did not discuss the evolution of nervous systems [45,46], the position of ctenophores as a sister group to all other animals implies two possible scenarios of neuronal origin: polygenesis or a single-origin (figure 2, left and right trees, respectively). It should be noted that both scenarios are also compatible with the two alternative hypotheses of the early animal phylogeny, in which either Ctenophora is the sister group to all Metazoa (figure 1) or Porifera is the sister to all other animals (see details in [14]).

I. *The single origin hypothesis* is consistent with the canonical and still most commonly accepted view of a single origin of neurons. Neuronal evolution was not analysed in the original *Mnemiopsis* genome manuscript [55]. However, in that study and a companion paper, it was proposed that the common ancestor of all animals had a nervous system, but that sponges and placozoans had lost their neurons [63]. ‘It appears that much of the genetic machinery necessary for a nervous system was present in the ancestor of all extant animals. This pattern suggests that a less elaborate nervous system was present in the metazoan ancestor and was secondarily reduced in placozoans and sponges’ [55, p. 1342]. Regarding the observed reduced representation of selected gene families in sponges and ctenophores, it was concluded that ‘this coinheritance suggests that the genetic machinery required for nervous system development might have been present in the pan-animal ancestor and, more controversially, that this ancestor might have had a not-so-simple nervous system’ [64, p. 1328]. The details supporting the monophyletic hypothesis were not provided.

By contrast, the systematic analysis of the *Pleurobrachia* genome and 10 other ctenophore transcriptomes, as well as extensive biochemical and functional studies, leads to a completely opposite conclusion: neural systems evolved more than once [28,65] (figures 1 and 5). We think that the hypothesis proposed by Ryan *et al*. [55] and Rokas [64] of the secondary loss of neural systems in sponges and placozoans is a very unlikely scenario for several reasons.

First, it is based on misinterpretations of the utilization of some individual genes as pan-neuronal or pan-synaptic markers, which is not accurate [40,66]. In fact, none of the ‘lines of evidence’ or genes that are specifically reported as factors ‘uniting the nervous systems of ctenophores, cnidarians and bilaterians’ can be used as universal neuronal markers (see detailed discussions in [40,66]; electronic supplementary material, table S1 and in the next paragraph). For the hypothesis of a ‘single origin of neurons’ to be valid, a cohort of neural-specific genes or regulatory non-coding regions that are found exclusively in ctenophores, cnidarians and bilaterians, but not in phyla without neurons (Placozoa and Porifera), must be specified. Next, these neural-specific genes must be localized in ctenophore neurons and/or control ctenophore neurogenesis. Such lines of evidence do not currently exist (see also details in [40]). *In situ* hybridizations were performed during *Mnemiopsis* development, suggesting the presence of distinct ‘neuronal’ genes that are shared between ctenophores and other animals, but their neural specification and co-localization has never been analysed [67]. By contrast, there are multiple examples of either the absence of bilaterian neurogenic genes in ctenophores or the lack of expression of presumed bilaterian/cnidarian ‘neuronal’ markers (e.g. ELAVs or Musashi) in ctenophore neurons [28,40,66], consistently with the polygenesis hypothesis.

Second, the single-origin hypothesis implies at least three events of the loss of ancestral neural systems in sponges, placozoans and ctenophores (figure 2, right) as well as the massive loss of many genes and molecular components involved in neurogenesis and synaptic functions from the urmetazoan. If correct, it would be critically important to identify the genes that were supposedly lost in sponges and placozoans (but preserved in ctenophores) that led to the loss of neurons and synapses. We believe that this massive functional neuronal/synaptic loss is also unlikely, because there is not a single example of a loss of neural systems in any non-parasitic animal lineage [68]. Indeed, according to the monophyletic hypothesis, it should be assumed that the common ancestor of ctenophores shared the same transmitters and neurogenic genes as the extant members of the Porifera, Placozoa and bilaterian–cnidarian clades. During the course of evolution, for unknown reasons, ctenophores subsequently lost most of these genes and replaced them, including most of the low-molecular-weight transmitters, with new types of signalling molecules (figure 2, right tree). However, ctenophores are active marine predators with complex behaviours [69,70]. It is unclear what past events or factors of natural selection would favour the loss of such complex transmitter signalling and neurogenic machinery in free-living (not parasitic!) ctenophores [14,28], particularly when such cellular machinery is highly conserved in other eumetazoan lineages. In any of these speculative cases, the single origin hypothesis still implies independent recruitment of other molecular components that are involved in neural and synaptic functions, a situation that still reflects the extensive parallel and convergent evolution of neural organization in ctenophores [66].

Third, in the originally published monophyletic reconstructions [64,71], any neural system was considered as a single character. This is also not accurate, owing to the enormous heterogeneity of neuronal populations within any single species and across phyla. It is more appropriate to discuss neuronal origin(s) in terms of the evolution of distinct cell lineages [72] with potentially different genealogies/origins and to implement interdisciplinary approaches to rigorously test sister relationships among them. Unless it is proved that all neuronal cell lineages across phyla share the same gene regulatory developmental programme(s) and unique markers confirming their homology, we should view a neuron as a functional but not a genetic character [14,66].

II. *The polygenesis* (*convergence*) *hypothesis* (figures 1 and 2, left) assumes multiple origins of neurons across phyla [13]. In 2012, it was proposed that neurons, as secretory cells, evolved independently in ctenophores [27]. Do ctenophores have the same machinery for neuronal/molecular signalling as other organisms with neural systems, such as bilaterians and cnidarians? If so, this would support a single origin hypothesis. If not, it would be consistent with the independent origins of neurons in ctenophores and cnidarians/bilaterians.

Subsequent genomic, functional and metabolomic studies confirmed that ctenophores recruit a largely *different subset of neurogenic and secretory molecules* for interneuronal signalling [28,40,66,73]. Thus, the dramatically different chemical language reported in ctenophore nervous systems [28,66] reflects their extensive parallel and convergent evolution. A corollary of the independent origin of neurons from ancestral secretory cells is the *convergent evolution of synapses* and synaptic signalling. The eukaryotic exocytosis machinery (for the formation and release of secretory vesicles) can independently recruit various uptake systems (transporters) and receptor proteins. Subsequently, this process would lead to the development of a number of cell lineages with various classes of secretory specificity, the ancestors of extant synaptic and neuronal classes. It could also result from a transition from temporal-to-spatial cell differentiation programmes following the origin of Metazoa [74].

We recently uncovered that Horridge discussed similar ideas in 1974 as a result of his ultrastructural studies of ctenophore synapses, which caused him to consider the physical constraints of synaptic transmission in general. ‘Therefore, the anatomy of a synapse follows from the physical properties of its components, so that *anatomical synapses of similar appearance in different groups of animals could be the result of convergent evolution*’ [75, p. 466] (emphasis ours).

It should also be noted that at the same time, in 1974, Sakharov [76] explicitly stated the idea of the polygenesis of neurons, asking why there are so many transmitters. In his reconstruction, the diversity of transmitters is a consequence of the independent origins, even in Bilateria, and parallel evolution of different neuronal lineages that preserved their ancestral type of transmitter specificity [24,25,76–78]. Sakharov also viewed transmitter specificity (or equivalently, secretory specificity) as one of the most evolutionarily conserved characteristics of neurons. He was the first person to use the apparent conservation of transmitter phenotypes, and he adjusted Remane's criteria (‘positional’, ‘structural’ and ‘transitional’ [79,80]) to identify individual homologous neurons across gastropod molluscs [76]. Simply put, his hypothesis states that neurons with different transmitter/secretory specificity are different because they had different origins and genealogies. Perhaps the most intriguing corollary of Sakharov's neuronal polygenesis is the scenario in which the serotonergic and dopaminergic neurons, for example, might have evolved from different pre-neuronal/ancestral cell lineages, and a brain is a mosaic of different cell lineages.

The different molecules themselves might be independently recruited to support intercellular and interneuronal signalling functions. Even the terminology should be adjusted, because there are many cases when classical (neuro)transmitters are present in non-neuronal cells. Some cell lineages that use various transmitters might or might not share genealogies with certain neuronal cell types. For example, the majority of serotonin in the human body is located in genetically and developmentally different mast cells rather than in neurons [81–83]. Similarly, acetylcholine is not a ‘pure’ neurotransmitter—it is highly abundant in immune T cells [84] as one of many of examples of the non-neuronal functions of transmitters that are independently recruited for long-distance signalling and systemic functions. Thus, a more correct term would be a *transmitter* (as a chemical mediator of signalling between cells) rather than would be a *neurotransmitter* (a term that refers to a more specialized situation in which the chemical signalling occurs only between neurons or neurons and their effectors). The genealogy of transmitters is an exciting, promising, but not well-developed field of research. The modularity and redundancy of certain cellular and molecular functions in neurons and other cell types should also be considered in future reconstructions.

We favour the polygenesis hypothesis, because many components of the molecular machinery controlling (i) neurogenesis, (ii) transmitter synthesis, (iii) receptor pathways, and (iv) ‘pre- and postsynaptic’ genes (including neuroligins and neurexins) are also absent in the unicellular eukaryotes recognized as sister groups of animals. Therefore, we hypothesize that the common ancestor of all animals (Urmetazoa) was an organism without defined neurons and synapses. Therefore, the ancestral (nerveless) state is still currently preserved in both extant sponges and placozoans—the lineages which, independently of ctenophores, developed chemical intercellular communications with unique subsets of secretory cells [85,86] and behaviours [87–89]. Some of these ancient and versatile molecular complexes might have been subsequently recruited into the neural systems of cnidarians and bilaterians.

We favour the polygenesis hypothesis, because ctenophores lack the majority of ‘neuron-specific’, neurogenic bilaterian/cnidarian genes, and because the genes of the ‘classical’ neurotransmitter pathways are either absent or, if present, are not expressed in neurons [28]. For example, we found that *GABA immunoreactivity is localized in muscles,* but it was not detected in any of the 5000–7000 ctenophore neurons [28,66]. The majority of the known low-molecular-weight transmitters (e.g. acetylcholine, serotonin, histamine, dopamine, noradrenaline and octopamine) are absent in ctenophores, as determined by direct microchemical and pharmacological experiments [28]. The surprising absence of canonical transmitters is paralleled by the development of unique/ctenophore-specific peptide-like molecules without any recognized homologues in other metazoans [28,40]. All of these findings are consistent with the hypothesis that the ctenophore neural systems evolved independently from those in other animals.

## 4. Potential criticisms of the hypothesis of the convergent evolution of neurons and synapses in ctenophores

Three recent papers [57,58,71] have reported several points that, according to the authors, might favour the hypothesis of single origin of neurons. We will briefly summarize these studies and our arguments.

(1) The proposed position of ctenophores is the result of tree construction artefacts (e.g. long-branch attraction (LBA), sampling, phylogenomic models, etc.), and sponges are the sister to all other lineages [58]. These are very relevant concerns. Currently, novel data with more species and different models [59,60] strongly support the Ctenophora-first hypothesis (figure 1), and no evidence for LBA was obtained. In addition, phylogeny is an important feature, but it less relevant on its own [14,90].
(2) The presence of selected genes known as neuronal fate and patterning genes (e.g. Lhx/LIM, Hes, Bhlh, Sox, NKL and Tlx) [71] and neuronal markers (e.g. ELAV, Musashi) might unite the nervous systems of ctenophores, cnidarians and bilaterians [55,57]. Although these genes are present in the ctenophore genomes, they cannot be considered as neuronal or pan-neuronal markers in ctenophores because they are expressed in many other cell types and in developmental stages when no neurons are present. Importantly, the specific co-localization of these markers with neurons has not been shown. In addition, homologues of these genes exist in nerveless sponges (electronic supplementary material, table S1), which further suggests that *pan-neuronal genes have not currently been identified across all metazoans* [40].
(3) ‘The presence of many components critical for synaptic function in bilaterians (e.g. Cadherin, Ephrin, Pmca, mGluR, Magi, Pkc, Citron, Spar, Dlg, Syngap, Gkap, Nos, Lin-7 and Pick1)’ [71]; and there are uncertainties about RIMs, ELKS [57], and the interpretation of gap junctions proteins as neuronal/synaptic markers. These genes are equally critical for many other, non-neuronal functions, and, in bilaterians, they are also expressed in diverse, non-neuronal tissues. Therefore, they cannot be considered to be unique synaptic markers, even in bilaterians. In addition, many of these genes are present in the genomes of unicellular organisms, including *Monosiga* and *Capsaspora*, as well as in other non-metazoan eukaryotes (e.g. NOS, see also the electronic supplementary material, table S1). In ctenophores, these genes are also expressed during developmental stages when no neurons and synapses are present [91]. Currently, we cannot identify specific and pan-synaptic genes for all Metazoa with synapses [40].

Recently, ctenophore neurons have been systematically mapped using tyrosinated *α*-tubulin antibodies in both developing and adult *Pleurobrachia* [28,91,92]. We estimated that approximately 5000–7000 neurons are present in the subepithelial and mesogleal neural nets of *P. bachei* ([28], L. Moroz and T. P. Norekian 2014, unpublished observations), providing a good reference platform to study the co-localization of candidate neurogenic and synapse-specific genes in enigmatic ctenophore neurons. A systematic comparison of the expression of more than 100 bilaterian ‘neuronal’ and ‘synaptic’ orthologues [14,28,66,93,94] led us to a simple conclusion. To the best of our knowledge, we cannot assign the ‘pan-neuronal’ or ‘pan-synaptic’ tag to any single gene across all metazoan lineages [28,94]. Many of the proposed ‘pan-neuronal’ or ‘pan-synaptic’ candidate genes are also detected during development, before neuronal specification [28,94]. Of course, we do not exclude the possibility that some genes (and/or neuron-specific enhancers and other non-coding genome elements acting as master regulators) could be uniquely and differentially expressed in all metazoan neurons when more taxa are included in such analyses, but careful co-localization studies are needed.

## 5. Parallel evolution of ctenophore chemical signalling and synapses

The presence of chemical transmission in ctenophores is well supported by functional studies [69,70,75]. However, the ultrastructure of their synapses is remarkably different from those in the majority of metazoans. Structurally, the asymmetrical synapses in ctenophores are quite organized, forming a so-called presynaptic triad, with little or no development of presynaptic density elements [95–97]. Each presynaptic element contains a tripartite complex of organelles: a single layer of synaptic vesicles lining the presynaptic membrane, a cistern of agranular endoplasmic reticulum just above the row of vesicles, and one or more mitochondria. The postsynaptic density, however, is less prominent in ctenophore synapses [95].

To our surprise, we found no biosynthetic enzymes for known ‘neuro'transmitters (i.e. acetylcholine, serotonin, histamine, dopamine and octopamine), with the exception of glutamate and GABA. Importantly, the genomic predictions were experimentally validated using ultrasensitive capillary electrophoresis with attomolar limits of detection [28,94]. This suggests that the ctenophore nervous systems are remarkably distinct among metazoans in terms of their most fundamental characteristics—chemical neurotransmission. The potential signalling roles of small secretory peptides, ATP, glycine and nitric oxide (NO) can be expected (e.g. the presence of genes encoding the relevant synthetic enzymes and receptors; table 1), but their functions as interneuronal messengers in ctenophores must be confirmed experimentally.

Currently, the only molecules identified as candidates for neuromuscular transmission are l-glutamate and perhaps l-aspartate [28]. Mistakenly, the ‘sensitivity to some classical neurotransmitters (e.g. l-glutamate)’ was interpreted as a characteristic uniting the nervous systems of ctenophores, cnidarians and bilaterians [71]. Conversely, the deeper analysis of the relevant pathways illustrates the extensive parallel evolution of the glutamate synthetic, transport and receptor pathways in ctenophores.

## 6. Unique features of glutamatergic signalling in ctenophores

l-Glutamate has the highest affinity to induce action potentials, elevate [Ca^2+^] in ctenophore smooth muscle cells and cause muscle contractions, whereas other transmitter candidates are ineffective, even at concentrations up to 5 mM [28]. To our surprise, we found that ctenophores possess an enormous diversity of all components involved in glutamate signalling, including the synthetic, transport, receptor and inactivation pathways.

**Figure 3.**
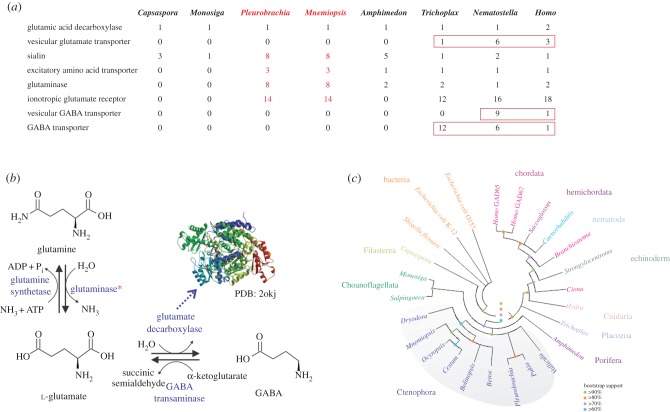
Glutamate metabolism and processing. (*a*) Table of pertinent genes used in glutamate metabolism and processing. Red numbers indicate expansions of those families in ctenophores. (*b*) The predicted metabolites, cofactors and enzymes of glutamate metabolism in ctenophores. The asterisk indicates the expansion of glutaminases in ctenophores. Crystal structure of GAD 67, PDB: 2okj is indicated as an insert [96]. (*c*) Genealogical relationships between the predicted glutamate decarboxylase (GAD) enzymes in several ctenophore species compared with prokaryotic and eukaryotic GAD enzymes. The ctenophore GAD enzymes share highest identity to each other forming a distinct branch (highlighted in grey). Maximum-likelihood trees were constructed based on the pyridoxal-dependent decarboxylase conserved domain (PF00282) amino acid sequences of the identified GADs. See the electronic supplementary material for methods.

Specifically, we cloned and localized fourteen ionotropic glutamate receptors (iGluRs) and eight sialin-like glutamate transporters in *P. bachei* [28,93] (see figure 3*a* for relevant genes). We showed an unprecedented diversity of iGluRs in ctenophores, forming a distinct branch on the genealogical tree for metazoan iGluRs topology [28]. Thus, iGluRs might have undergone a substantial adaptive radiation in Ctenophora as also evidenced by unique exon/intron organization for many subtypes [28]. Importantly, *ctenophore iGluRs could not be classified in the terms of vertebrate iGluR families* (i.e. *α*-amino-3-hydroxy-5-methyl-4-isoxazolepropionic acid, kainate, delta or *N*-methyl-d-aspartate (NMDA), but our analysis suggests that ctenophore iGluRs could be related to an ancestral family including earlier NMDA-type receptors (see Extended Data fig. 7 in [28]), and potentially might be activated or blocked by other endogenous ligands (e.g. glycine in addition to l-glutamate).

There are eight glutaminases—the enzymes that convert glutamine to glutamate. The glutamine aminohydrolase, or l-glutaminase (GLS), converts glutamine to glutamate (figure 3*b*). Glutamate is also involved in bioenergetics via the tricarboxylic acid cycle and the production of ATP, and ADP is a strong activator of the GLS enzymes [99,100]. *Pleurobrachia bachei* and *M. leidyi* have eight and nine GLS enzymes, respectively. In contrast, most metazoans have one GLS enzyme, and humans have two GLS enzymes (figure 3*a,b*). The apparent expansion of the glutamate synthetic enzymes in ctenophores might be related to the observed diversification of glutamate signalling and the high bioenergetic demands of the extensive development of ciliated structures and associated behaviours [70], with multiple examples of lineage-specific diversification events [28,40]. Thus, although ctenophores (including *Pleurobrachia*, *Bolinopsis* and *Mnemiopsis*) have muscle sensitivity to l-glutamate, their synthetic, transport, receptor and inactivation pathways are notably distinct from those identified in cnidarians and bilaterians.

## 7. Gamma-aminobutyric acid synthesis and localization in ctenophores

GABA is the downstream product of glutamate metabolism and inactivation. Glutamate decarboxylase (GAD), a GABA synthetic enzyme (figure 3*b,c*), is widely distributed across both prokaryotes and eukaryotes [101]. GAD is related to the group II pyridoxal-dependent decarboxylase enzymes (PF00282), which include the glutamate, histidine, tyrosine and aromatic-l-amino acid decarboxylases. Previous studies have shown that these pyridoxal-dependent decarboxylases have evolved in parallel along multiple lineages [101,102]. The predicted ctenophore GAD enzymes have all of the critical amino acids essential for GAD enzyme function, as defined by the crystal structure [98]. The functional GAD activity in ctenophores is supported both by immunohistochemical visualization of GABA in muscles [66] and by direct microchemical detection of GABA in the extracts from different ctenophore organs [28].

Surprisingly, in ctenophores, GABA is localized only in muscles and not in neurons [28,66]. In addition, there were no observable pharmacological effects of GABA on the *Pleurobrachia* behaviours and major effector systems, including cilia, muscles and colloblasts [28]. These observations suggest that GABA functions as metabolic/bioenergetic intermediate, and as a possible mechanism to inactivate glutamate. A product of GABA metabolism can itself be a usable source of energy in ctenophore muscles. Indeed, GABA transaminase, which is also found in *Pleurobrachia*, is the enzyme that catalyses the conversion of GABA back into succinic semi-aldehyde and glutamate, following the formation of succinic acid, which enters the citric acid cycle—the universal aerobic bioenergetics pathway (figure 3*a,b*). By contrast, GABA is specifically localized in selected neuronal populations of both bilaterians and cnidarians, suggesting that it was subsequently co-opted in evolution as a *neuro*transmitter.

In summary, our data suggest that the observed diversification of glutamate processing is coupled with an enormous functional complexity of glutamate signalling in ctenophores. Interestingly, the expression levels of the majority of genes associated with glutamate processing were substantially increased on day three of *Pleurobrachia* development, when the first neurons appeared [28,91]. Thus, the neurogenesis and the fate specification of glutamatergic neural signalling might be mechanistically coregulated by the orchestrated expression of all of the molecular components that support l-glutamate synthesis (glutaminases), its inactivation (transporters/sialins), and its reception (iGluRs). Owing to a possible ‘deficiency’ of other classical transmitters, ctenophores might have ‘taken advantage’ of the versatile l-glutamate molecule and further developed an unprecedented complexity of glutamatergic systems to accommodate their complex behaviours. As a result, extant ctenophores possess one of the most unusual and molecularly diverse complements of glutamate signalling pathways of all animals studied to date. This is also an illustrative example of the extensive parallel evolution of the nervous system.

## 8. Convergent and parallel evolution of electrical synapses in Metazoa

**Figure 4.**
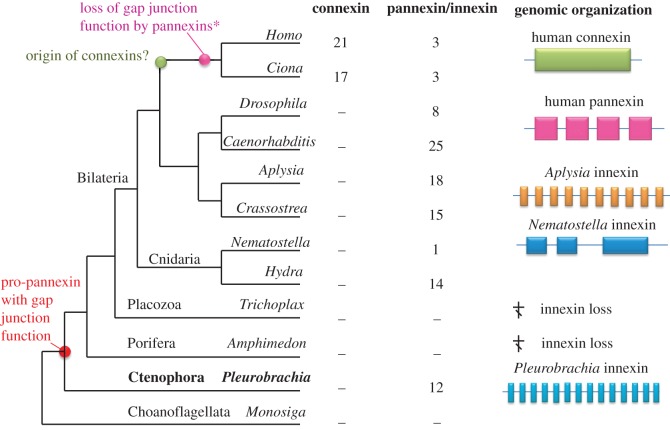
Convergent evolution of gap junction proteins. Connexins are a chordate-specific family of genes encoding gap junction proteins; they share no identity to the pannexin/innexin (PANX/INX) family. However, both families have the same membrane topology and perform the same function as electrical synapses and membrane pores. The table shows the complement of connexins and pannexin genes in different metazoans with sequenced genomes. The right part illustrates examples of the organization of representative genes. *Pleurobrachia PANX/INX* genes have the largest number of exons compared with all other metazoans.

Historically, pore-forming proteins are broadly divided into two groups: connexins (only identified in chordates) and pannexins, which are present in both chordates (including tunicates and vertebrates) and invertebrates (figure 4). Before their discovery in humans [103], the invertebrate pannexins were initially named innexins—i.e. the invertebrate counterpart to gap junctions. Despite the lack of apparent sequence similarity with connexins, the innexin/pannexin (PANX/INX)-type gap junctions share the same overall membrane topology as connexins. Each pore is composed of four transmembrane regions in which six individual subunits form a channel or ‘innexon’ in the plasma membrane [104]. Two opposing innexons on interacting cells form a functional electric synapse. All gap junctions (connexins and pannexins) mediate fast electrical coupling between cells, which can be symmetrical or asymmetrical, allowing directional information processing. Importantly, pannexins form gap junctions with electrophysiological and pharmacological properties that are distinct from connexins [105]. Thus, these two dissimilar families of proteins represent a perfect example of the convergent evolution of electrical synapses.

We did not find any gap-junction orthologues in choanoflagellates or other eukaryotic groups, suggesting that pannexins/innexins are metazoan innovations [103,106,107]. In addition to metazoans, *PANX/INX* genes have been found in a few viruses, possibly as a result of lateral gene transfer between a host and its intracellular parasites [108]. For example, the endoparasitic wasp *Campoletis sonorensis* transmits a polydnavirus to its caterpillars during egg laying [108]. The sequenced ichnovirus (IV) genome has significant similarity to some parts of the wasp genome, including the presence of *PANX/INX* genes [108]. Predictably, these IV *PANX/INX* are closely related to the pannexins from arthropods (electronic supplementary material, figure S1).

The pannexin/innexin superfamily has a highly mosaic representation across metazoans, with several examples of gene gains and gene losses. There is no conservation in the exon–intron organization across pannexins. For example, only three *PANX* genes have been identified in mammals, all of which have four to five exons. The *Drosophila* genome encodes eight *PANX/INX* genes with three to eight exons [109]. Twenty-five *PANX/INX* genes are present in the *C. elegans* genome, with 3–11 exons [110]. In *Aplysia californica*, 18 *PANX/INX* genes have been identified, with 1–10 exons (figure 4). Non-bilaterian metazoans have the most unusual complements of *PANX/INX* genes. The *A. queenslandica* [30] and *T. adhaerens* [29] genomes and the publically available sponge transcriptome data [111] have no identified pannexins.

Cnidarians appear to have both losses and expansions of the *PANX/INX* genes. The hydrozoan *Hydra magnipapillata* has at least 19 pannexins [112,113]. By contrast, the anthozoan *Nematostella vectensis* has only one recognized *PANX/INX* gene (figure 4 and electronic supplementary material, figure S1), which may be involved in the electrical coupling between blastomeres in embryos [114]. However, no *PANX/INX* genes have been detected in the genomes of three other anthozoans, *Aiptasia* [115], *Acropora digitifera* [116] and *Stylophora pistillata*. There are also no identified *PANX/INX* genes in the scyphozoan *Cyanea capillata* (based on our transcriptome profiling). It is still unknown whether the cubozoans have *PANX/INX* genes. Gap junction proteins (both innexins and connexions) tend to be present in multiple copies in almost all metazoans, with the exception of *Nematostella*. Interestingly, the predicted *Nematostella* PANX/INX protein is clustered at the base of the chordate clade (electronic supplementary material, figure S1; this has also been observed by Abascal & Zardoya [106]). Given these observations, it has been suggested that there has been a horizontal transfer of a *PANX/INX* gene from an ancestral chordate to *Nematostella* [106].

In contrast to the cnidarians, all sequenced ctenophores have an enormous diversity of electrical synapses or gap junctions [28], forming a distinct branch in tree topology (electronic supplementary material, figure S1). However, no connexins are encoded in the genomes of *Pleurobrachia* and *Mnemiopsis*, or in any of the other ctenophore transcriptomes analysed. In general, the ctenophore *PANX/INX* genes contain more exons than their orthologues in Hydrozoa and bilaterians (the number of exons varies from 1 to 14; figure 4). Although both *P. bachei* and *M. leidyi* have 12 *PANX/INX* genes in their genomes, only four of them form genealogical sister pairs between species (electronic supplementary material, figure S1), further suggesting the widespread lineage-specific radiation and parallel evolution of this family.

**Figure 5.**
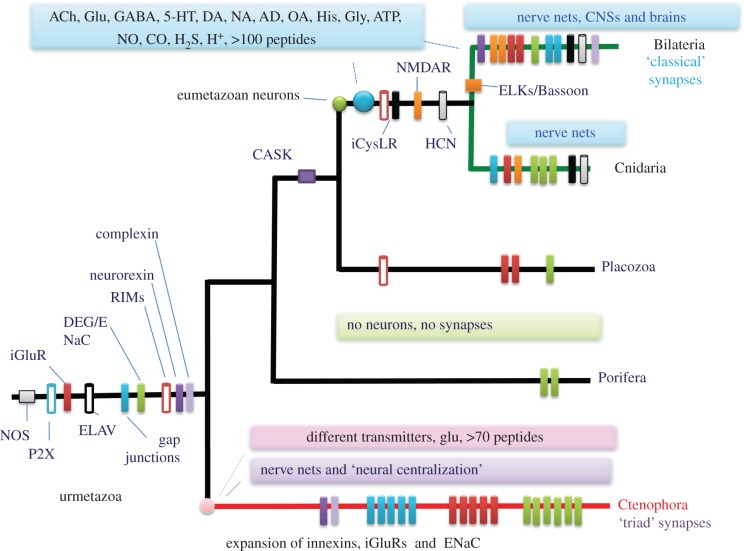
Key molecular innovations underlying evolution of neural organization in ctenophores and the cnidarian/bilaterian clade (modified from Moroz *et al*. [28]). Bars indicate the presence or relative expansions of selected gene families in all basal metazoan lineages from the inferred urmetazoan ancestor. The data suggest that sponges and placozoans never developed neural systems, or, highly unlikely assuming the presence of neuronal organization in the urmetazoan ancestor, sponges and placozoans lost their nervous systems. Either hypothesis points towards extensive parallel evolution of neural systems in ctenophores versus the Bilateria + Cnidaria clade. See text for details.

Functional analysis of the electrical synapses in ctenophores is in its infancy [70,117]. Interestingly, the *PANX/INX* genes are one of the most highly expressed transcripts in the adult aboral organ of *P. bachei* (figure 5*d*; [28]), but they are also expressed in the combs and conductive tracts and in the neuron-like subepithelial cells. Gap junctions have previously been identified in the ciliated grooves by electron microscopy [95], which run from the aboral organ to the first comb plate of each comb row and through the endoderm of the meridional canals [117]. These data suggest that a significant fraction of the synaptic transmission in the conductive tracts and the aboral organ is electrical. In addition to conducting electrical synapses, gap junctions also mediate mechanoreception [118] and the direct exchange of small molecules among neighbouring cells by forming channel aggregates in the plasma membrane without any synapses to release ATP or other molecules [119–123]. This possibility might explain the functional significance of the expression of selected types of PANX/INX during early *Pleurobrachia* development (figure 4*a*; [28]).

In summary, gap junction proteins are wonderful illustrations of the convergent evolution and independent lineage-specific diversification of synaptic organization. The chordate-specific connexins share no sequence identity with the pannexin-type gap junctions but have a similar membrane topology and perform the similar functions. Interestingly, some chordate pannexins have lost their gap junction functions [124]; therefore, the expansion of the connexin family in this lineage, with more than two dozen members, provides a complementary solution to support the diversity of electrical synapses in human brains, muscles and secretory tissues. The pannexin-type gap junctions may have originally evolved in the common metazoan ancestor to mediate the release of ATP or other metabolites to support intercellular communications during early development and in multicellular coordination. This might provide a foundation for the independent recruitment of pannexins and connexins into neuronal functions over the course of ctenophore, cnidarian and chordate evolution. The cladistic analyses suggest that *Amphimedon*, *Trichoplax* and some cnidarians lost their recognized gap junction proteins, but the significance of this type of gene loss is unclear. It is also possible that other classes of proteins can form functional gap junctions in these metazoan lineages indicating that the early evolution of pore-forming proteins is more complex than was previously anticipated.

## 9. Conclusion

Complementary studies [14,28,40,66] have revealed a number of unique features in the molecular organization of ctenophore signalling (figure 5), which is consistent with the hypothesis of the convergent evolution of neural and integrative systems. The most remarkable trait of Ctenophora is the apparent absence of the majority of the conventional low-molecular-weight transmitter systems. This is paralleled by the considerable development and diversification of the synthetic, uptake and receptor components of glutamate signalling, with extensive lineage-specific adaptations within the clade. Given the current placement of ctenophores as the sister group to other animals [60], the most plausible scenario suggests that acetylcholine, serotonin, histamine, dopamine, octopamine and GABA were recruited as transmitters in the common cnidarian/bilaterian ancestor. We believe that GABA originally evolved as a passive product of the inactivation of glutamate and as a bioenergetically important intermediate, as in extant ctenophores. Later in the cnidarian and bilaterian lineages, GABA was recruited into neuronal signalling as a synaptic messenger molecule. A similar scenario for the independent recruitment of NO in neuronal signalling can also be observed in various lineages of cnidarians and bilaterians [125–127]. It may well be that all other classical low-molecular-weight transmitters originally evolved as metabolic intermediates and non-neuronal, in part injury related [128] signalling molecules in early animals. This process was followed by multiple co-option events in different secretory cells, including the various (proto)neuronal cell lineages. The roles of the different transmitters in early embryonic development [129–137] might partially reflect these ancestral functions of the vast diversity of signalling molecules, from glutamate to small secretory peptides.

The origin and subsequent evolution of the neural systems in the ctenophore lineage occurred independently from those in all other animals (figures 1 and 5). Such processes frequently used a distinct array of ‘available’ intercellular messengers, including secretory peptides, which not only are unique for these groups, but also are undergoing very rapid evolution in ctenophores. ATP and related purines, glycine, nitric oxide, carbon monoxide and hydrogen sulfide might also be used as intercellular messengers [28,40], but with multiple ctenophore-specific innovations (figure 5). The predicted pre- and postsynaptic gene complements in *Pleurobrachia* and *Mnemiopsis* have reduced numbers of components compared with those of cnidarians and bilaterians (table 1). As a corollary of the hypothesis of the independent origins of neurons, our analyses suggest that both electrical and chemical synapses evolved more than once (figure 4).

## Supplementary Material

Methods

## Data accessibility

All raw data from all genomic for *P. bachei* are available at NCBI: SRA project: SRP001155. All raw data from transcriptomic data of all ctenophores analysed are available at NCBI: SRA project: SRP000992. All raw data were assembled and annotated on University of Florida Database: http://neurobase.rc.ufl.edu/. All *P. bachei* gene models used in the analysis were published and their description can be found at [2]. *M. leidyi* gene models were obtained at http://research.nhgri.nih.gov/mnemiopsis/jbrowse/jbrowse.cgi. The accession numbers for sequences used in all the analyses are listed in the electronic supplementary material, table S1.
