# Supplementary material for: Independent origins of neurons and synapses: insights from ctenophores
Source: Philos Trans R Soc Lond B Biol Sci. 2016 Jan 5;371(1685):20150041. doi: 10.1098/rstb.2015.0041 (PMC4685580; doi:10.1098/rstb.2015.0041)
Supplement: Methods [file rstb20150041supp1.pdf]

Supplementary Materials

All molecular methods performed, including cloning and *in situ* hybridization, have been described in Supplementary Information [1]. The construction of the RNA-seq libraries has been described [1]. All molecular methods performed including cloning and expression analysis have been described elsewhere [1]. All Pleurobrachia RNA-seq libraries were constructed as reduced representation (one read per one transcript) [1]. Thus, we report a relative level of expression (quantification) as a normalized frequency, where the number of reads that mapped to a specific transcript is divided by the total number of reads in a particular sequencing project then multiplied by a million to give Transcripts per Million or a TPM value.

Maximum likelihood trees were constructed based on amino-acid sequences. Alignments of all orthologs were performed with either ClustalX2 or Muscle then, if appropriate, either trimmed manually or trimmed using GBlocks for further comparative analysis of individual gene families. Pfam composition, Gene Ontology, and KEGG (Kyoto Encyclopedia of Genes and Genomes) were used to further validate orthologs. Once alignments were obtained, basic gene trees were constructed in MEGA 5. The molecular phylogenetic analysis used the Maximum Likelihood (ML) method based on the Whelan and Goldman (WAG) matrix-based model. The bootstrap consensus tree was inferred from 100 replicates. All positions containing gaps and missing data were eliminated. The percentage of trees in which the associated taxa clustered together is shown next to the branches. The tree is drawn to scale, with branch lengths measured in the number of amino acid substitutions per site. Larger phylogenetic analyses were conducted using ML in RAxML 7.2.7. For ML analyses, the CAT +WAG + F model was used. Topological robustness (i.e., nodal support) for all ML analyses was assessed with 100 replicates of nonparametric bootstrapping more details on methods can be found in [1].

All BLAST searches were on publically available databases including species-specific genome browsers and used an E-value $\leq 10^{-4}$ for cut-off values. All the accession numbers for sequence used in this study are listed below. All raw from all genomic data for *Pleurobrachia bachei* is available at NCBI: SRA Project: SRP001155. All raw data from transcriptomic data of all ctenophores analyzed is available at NCBI: SRA Project: SRP000992. All raw data was assembled and annotated on University of Florida Database: http://neurobase.rc.ufl.edu/. All *P. bachei* gene models used in the analysis were published and their description can be found at [2]. *M. leidyi* gene models were obtained at http://research.nhgri.nih.gov/mnemiopsis/jbrowse/jbrowse.cgi.

All *Amphimedon_queenslandica* data came from http://metazoa.ensembl.org/Amphimedon_queenslandica

Accession numbers for data used in phylogenetic analysis:

GAD tree

**Species**	**Database #/Accession #**
Mnemiopsis leidyi	ML234525a
Pleurobrachia bachei	2652796/2669193
Beroe abyssicola	346791
Bolinopsis infundibulum	1206719
Cestum veneris	1314307
Dryodora glandiformis	292886
Ocyropsis	1064711
Pukia falcata	13065841
Vallicula multiformis	372068
Capsaspora owczarzaki	EFW47217.1
Monosiga brevicollis	XP_001742762.1
Salpingoeca rosetta	XP_004993273.1
Shigella flexneri VA-6	EGK16006.1
Escherichia coli str. K-12	NP_417974.1
Escherichia coli	P69908.1
Branchiostoma floridae	EEN48152.1
Ciona intestinalis	NP_001027785.1
Saccoglossus kowalevskii	XP_002740674.1
Strongylocentrotus purpuratus	XP_001186613.1
Caenorhabditis elegans	AAD19958.1
Hydra magnipapillata	XP_002166171.1
Trichoplax adhaerens	XP_002116563.1
Amphimedon queenslandica	XP_003389906.1
Homo sapiens GAD67	NM_000817
Homo sapiens GAD65	NP_000809.

Pannexin/Innexin tree

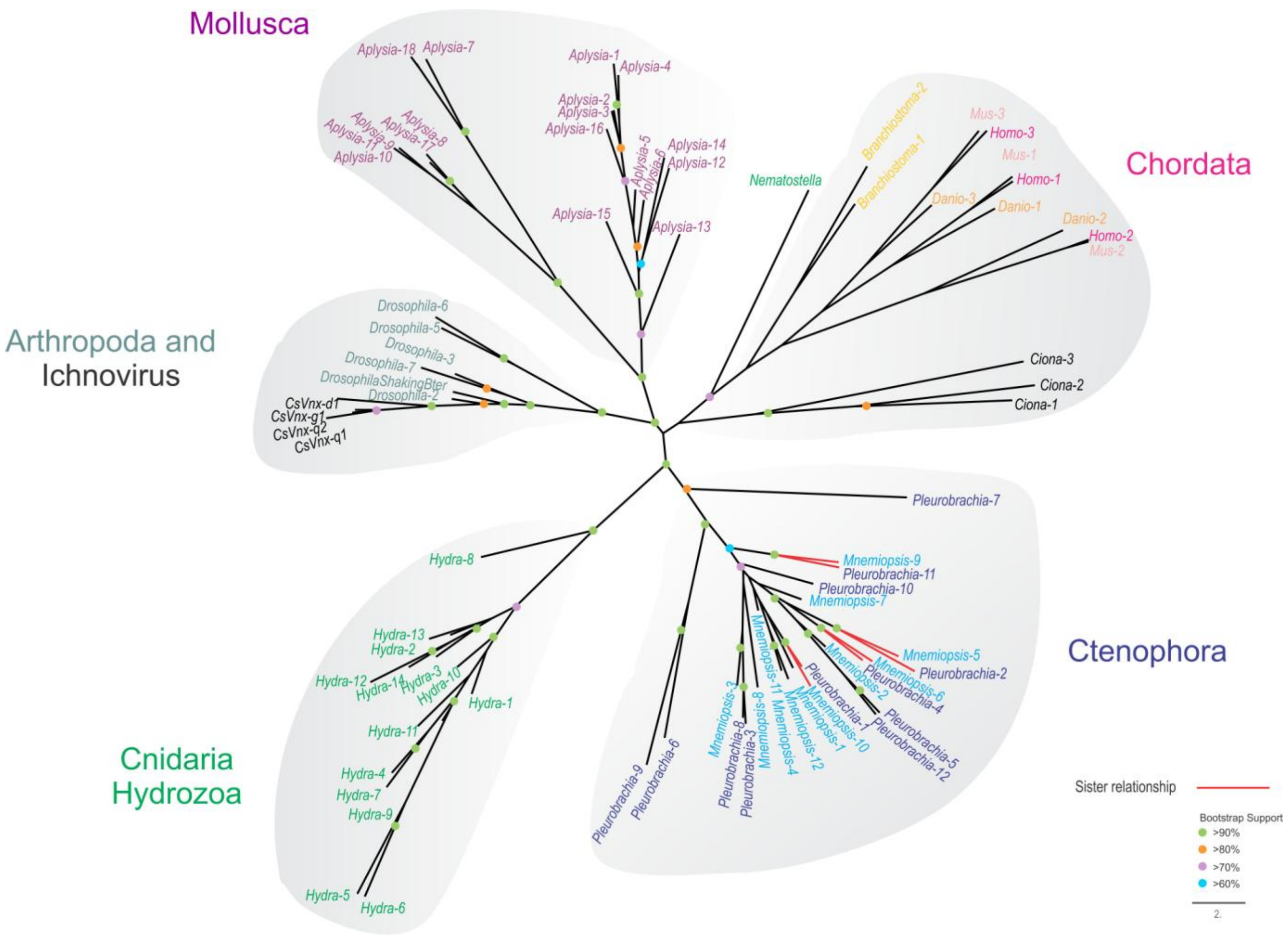

Figure 1S. Genealogical relationships among the genes encoding pannexin/innexin (PANX/INX) family in metazoans. The PANX/INX family shows lineage-specific diversification in several major metazoan clades. Predicted Ctenophore PANX/INX proteins also share highest identity to each other forming a distinct branch on the tree topology, but there are only four sister-type relationships between Pleurobrachia and Mnemiopsis, red line. See Supplementary material for methods.

**Species**	**Database #/accession #**	**Name in tree**
Mnemiopsis leidyi	ML036514a	ML036514a
Mnemiopsis leidyi	ML07312a	ML07312a
Mnemiopsis leidyi	ML078817a	ML078817a
Mnemiopsis leidyi	ML129317a	ML129317a
Mnemiopsis leidyi	ML218922a	ML218922a
Mnemiopsis leidyi	ML223536a	ML223536a
Mnemiopsis leidyi	ML25993a	ML25993a
Mnemiopsis leidyi	ML25997a	ML25997a
Mnemiopsis leidyi	ML25998a	ML25998a
Mnemiopsis leidyi	ML25999a	ML25999a
Mnemiopsis leidyi	ML32831a	ML32831a
Mnemiopsis leidyi	ML47742a	ML47742a
Pleurobrachia bachei	sb|12261620|	Pb1
Pleurobrachia bachei	sb|12261985|	Pb2
Pleurobrachia bachei	sb|12262142|	Pb3
Pleurobrachia bachei	sb|12263189|	Pb4
Pleurobrachia bachei	sb|12263403|	Pb5
Pleurobrachia bachei	sb|12263714|	Pb6
Pleurobrachia bachei	sb|12263940|	Pb7
Pleurobrachia bachei	sb|12264048|	Pb8
Pleurobrachia bachei	sb|12266054|	Pb9
Pleurobrachia bachei	sb|12269606|	Pb10
Pleurobrachia bachei	sb|12266222|	Pb11
Pleurobrachia bachei	sb|12277377|	Pb12
Homo sapiens	NP_056183.2	*Homo-1*
Homo sapiens	NP_443071.2	*Homo-2*
Homo sapiens	NP_443071.3	*Homo-3*
Mus musculus	NP_062355.2	*Mus-1*
Mus musculus	NP_443191.1	*Mus-2*
Mus musculus	NP_766042.2	*Mus-3*
Danio rerio	NP_957210.1	*Danio-1*
Danio rerio	NP_001243570.1	*Danio-2*
Danio rerio	XP_001919861.1	*Danio-3*
Ciona intestinalis	XP_002124490.2	*Ciona-1*
Ciona intestinalis	XP_002119287.2	*Ciona-2*
Ciona intestinalis	XP_009858431.1	*Ciona-3*
Nematostella vectensis	XP_001623899.1	*Nematostella*
Branchiostoma floridae	XP_002604233.1	*Branchiostoma-1*
Branchiostoma floridae	XP_002585873.1|	*Branchiostoma-2*
Drosophila melanogaster	NP_001162684.1	*Drosophila-2*
Drosophila melanogaster	NP_524730.1	*Drosophila-3*
Drosophila melanogaster	NP_573353.2	*Drosophila-5*
Drosophila melanogaster	NP_572374.1	*Drosophila-6*
Drosophila melanogaster	NP_788872.1	*Drosophila-7*
Drosophila melanogaster	NP_728361.1	*Drosophila-8*

Campoletis sonorensis	AAO45828.1	*CsVnx-d1*
Campoletis sonorensis	AAO45831.1	*CsVnx-q2*
Campoletis sonorensis	AAO45830.1	*CsVnx-q1*
Campoletis sonorensis	AAO45829.1	*CsVnx-g1*
Aplysia californica	NP_001191577.1	*Aplysia-1*
Aplysia californica	NP_001191579.1	*Aplysia-2*
Aplysia californica	NP_001191578.1	*Aplysia-3*
Aplysia californica	NP_001191576.1	*Aplysia-4*
Aplysia californica	NP_001191595.1	*Aplysia-5*
Aplysia californica	NP_001191594.1	*Aplysia-6*
Aplysia californica	NP_001191616.1	*Aplysia-7*
Aplysia californica	NP_001191596.1	*Aplysia-8*
Aplysia californica	NP_001191461.1	*Aplysia-9*
Aplysia californica	NP_001191462.1	*Aplysia-10*
Aplysia californica	XP_005110953.1	*Aplysia-11*
Aplysia californica	XP_005100630.1	*Aplysia-12*
Aplysia californica	XP_005109439.1	*Aplysia-13*
Aplysia californica	XP_005110669.1	*Aplysia-14*
Aplysia californica	XP_005103400.1	*Aplysia-15*
Aplysia californica	XP_005104811.1	*Aplysia-16*
Aplysia californica	XP_005110954.1	*Aplysia-17*
Aplysia californica	XP_005101166.1	*Aplysia-18*
Hydra vulgaris	NP_001274699.1	*Hydra-1*
Hydra vulgaris	XP_002160488.1	*Hydra-2*
Hydra vulgaris	XP_002166931.2	*Hydra-3*
Hydra vulgaris	XP_004213297.2	*Hydra-4*
Hydra vulgaris	XP_004212712.1	*Hydra-5*
Hydra vulgaris	XP_004212713.1	*Hydra-6*
Hydra vulgaris	XP_012561363.1	*Hydra-7*
Hydra vulgaris	XP_004208200.2	*Hydra-8*
Hydra vulgaris	XP_002170241.2	*Hydra-9*
Hydra vulgaris	XP_002164718.2	*Hydra-10*
Hydra vulgaris	XP_012566780.1	*Hydra-11*
Hydra vulgaris	XP_002165350.1	*Hydra-12*
Hydra vulgaris	XP_012554059.1	*Hydra-13*
Hydra vulgaris	XP_002170247.1	*Hydra-14*

Table 1S

Gene	Description	*Capsaspora*	*Monosiga*	*Pleurobrachia*	*Mnemiopsis*	*Amphimedon*	*Nematostella*	*Nematostella*	*Homo*	Reference
Cadherin	Cell adhesion									[2, 3]
Profilin	Scaffolding									[4]
Spar	Signalling									[2, 3]
Dlg	Scaffolding									[2, 3]
Pkc	Enzyme									[2, 3]
PMCA	Ion transport									[2, 3]
Bhlh	Transcription factor									[2, 3]
Magi	Scaffolding									[2, 3]
Gkap	Signalling									[2, 3]
ELAV	RNA-binding									[4]
Pick1	Signalling									[2, 3]
SOXB	Transcription factor									[4]
CRIPT	Signalling									[2, 3]
LIM homeodomain	Transcription factor									[4]
Munc13	Scaffolding									[4]
mGluR	Receptive									[2, 3]
Lin-7	Scaffolding									[2, 3]
Citron	Signalling									[2, 3]
NOS	Signalling									[2, 3]
SynGap	Signalling									[2, 3]
Ephrin	Signalling									[2, 3]
RIMs	Scaffolding									[4]
ELK	Scaffolding									[4]

Representation of different gene families in five basal metazoan genomes (*Pleurobrachia*, *Mnemiopsis, Amphimedon, Trichoplax, Nematostella* and *Homo* as well as in genomes of two eukaryotic lineages (*Capsaspora* and *Monosiga*) know to be sister to Metazoa. These distribution indicate that many genes discussed in the literature as neuronal and synaptic markers present both in organisms without recognized neurons (*Amphimedon* and *Trichoplax*) and in non-metazoan eukaryotes – therefore they cannot be viewed as pan-neuronal or pan-synaptic genes and cannot be considered as an evidence for the presence of neural organization in the common ancestor of all animals – the urmetazoan. See main text for details.

References:

[1] Moroz, L.L., Kocot, K.M., Citarella, M.R., Dosung, S., Norekian, T.P., Povolotskaya, I.S., Grigorenko, A.P., Dailey, C., Berezikov, E., Buckley, K.M., et al. 2014 The ctenophore genome and the evolutionary origins of neural systems. *Nature* **510**, 109-114. (doi:10.1038/nature13400).

[2] Ryan, J.F. 2014 Did the ctenophore nervous system evolve independently? *Zoology (Jena)* **117**, 225-226. (doi:10.1016/j.zool.2014.06.001).

[3] Ryan, J.F., Pang, K., Schnitzler, C.E., Nguyen, A.D., Moreland, R.T., Simmons, D.K., Koch, B.J., Francis, W.R., Havlak, P., Smith, S.A., et al. 2013 The genome of the ctenophore Mnemiopsis leidyi and its implications for cell type evolution. *Science* **342**, 1242592. (doi:10.1126/science.1242592).

[4] Marlow, H. & Arendt, D. 2014 Evolution: ctenophore genomes and the origin of neurons. *Curr Biol* **24**, R757-761. (doi:10.1016/j.cub.2014.06.057).
